# THE DIRECT EFFECT OF COMMUNITY-LEVEL RISK FACTORS ON LUNG CANCER INCIDENCE IN FOUR NEW ENGLAND STATES

**DOI:** 10.1093/geroni/igad104.2375

**Published:** 2023-12-21

**Authors:** Taylor Jansen, Qian Song, Kristie Long-Foley, Elizabeth Dugan

**Affiliations:** University of Massachusetts Boston, Boston, Massachusetts, United States; University of Massachusetts Boston, Boston, Massachusetts, United States; Wake Forest University School of Medicine, Winston-Salem, North Carolina, United States; University of Massachusetts, Boston, Boston, Massachusetts, United States

## Abstract

Lung cancer is the most common cancer globally and 70% of diagnoses are among the 65+ population. This study identifies what town-level risk factors are contributing to geographic disparities in 65+ lung cancer in N=796 towns in Connecticut, Massachusetts, New Hampshire, and Rhode Island. Multiple spatial-based analyses (e.g., Moran’s I, Getis-Ord Gi*, mapping) were conducted before running a series of spatial regression models to determine the association between town-level risk factors (i.e., environmental exposures, respiratory conditions prevalence, education rates, smoke free legislation) and lung cancer incidence. Mapping and Moran’s I test suggested the 65+ rates of lung cancer in our sample were non-randomly distributed and clusters of high and low rates were present. A hot spot analysis was conducted in ArcMap 10.8 which identified several significant clusters of 65+ lung cancer. Spatial regression models showed that community rates of comorbid respiratory conditions (65+ asthma, COPD, and tobacco use disorder (TUD)) increased lung cancer incidence by 7%, while increasing ozone emissions were directly associated with a 10% increase in incidence. Comprehensive smoke free legislation coverage was significantly associated with lung cancer incidence, but no increasing or decreasing relationship was found. No effects were found for the percentage of 65+ with low education, or 60+ who were current or former smokers. Spatial regression models allow for results which are specific to the geographic sample used and this study suggests that comorbid respiratory conditions and ozone emissions are directly associated with increased rates of 65+ lung cancer incidence in our sample.

